# Phosphorylated tyrosine-containing proteins in primary lung cancer correlates with proliferation and prognosis

**DOI:** 10.1038/sj.bjc.6600327

**Published:** 2002-06-17

**Authors:** Y Gong, T Hirano, Y Kato, K Yoshida, Y Shou, T Ohira, N Ikeda, Y Ebihara, H Kato

**Affiliations:** Department of Surgery, Tokyo Medical University, 6-7 Nishishinjuko, Shinjuku-ku, Tokyo 160-0023, Japan; Department of Pathology, Tokyo Medical University, 6-7 Nishishinjuko, Shinjuku-ku, Tokyo 160-0023, Japan

**Keywords:** phosphorylated tyrosine-containing proteins, cell proliferating activity, histopathological differentiation, poor prognosis, lung cancer

## Abstract

To determine the usefulness of tyrosine phosphorylation in evaluating biological characteristics, we attempted to evaluate the relationship between the amount of phosphorylated tyrosine-containing proteins and clinicopathological factors, cell proliferation and outcome in non-small cell lung cancer. To evaluate phosphorylated tyrosine-containing proteins we used 96 surgically resected materials of non-small cell lung cancer and normal peripheral lung, while immunohistochemical evaluation was performed. Cell proliferating ability was evaluated using the labelling index of proliferating cell nuclear antigen-positive nuclear staining cells. There were statistically significant differences between the expression levels of phosphorylated tyrosine-containing proteins of normal and cancerous tissues (*P*<0.0001). Evaluations based on clinicopathological factors apart from histopathological differentiation, showed no statistically significant differences of phosphorylated tyrosine-containing proteins expression. However, phosphorylated tyrosine-containing proteins correlated with cell proliferation activity evaluated (*P_(Low, High)_*<0.0001; *P_(Low, Int)_* <0.0001; *P*_(*Int, High*)_<0.0001). Furthermore, non-small cell lung cancer cases with high expression and intermediate expression of phosphorylated tyrosine-containing proteins had a significantly shorter disease-free postoperative survival than those with low expression of phosphorylated tyrosine-containing proteins using log-rank analysis (*P_(Low, Int)_* <0.0028; *P_(Low, High)_*=0.0002). Furthermore, phosphorylated tyrosine-containing proteins expression level statistically contributed to disease-free survival in Cox's proportional hazard model. Therefore, phosphorylated tyrosine-containing proteins in non-small cell lung cancer tissues seem to reflect its biological malignancy, and this evaluation may be valuable for constructing the most appropriate therapeutic strategy.

*British Journal of Cancer* (2002) **86**, 1893–1898. doi:10.1038/sj.bjc.6600327 www.bjcancer.com

© 2002 Cancer Research UK

## 

Primary lung cancer is one of the most malignant solid tumours. In spite of current improvements in combined therapy, its incidence and mortality rates are still increasing in Japan. Since the clinical courses of lung cancer patients are extremely varied, and an understanding of the individual characteristics of the tumour could be important, and the evaluation of useful biomarker may be valuable for constructing the most appropriate therapeutic strategy.

Recently, extensive studies have been performed to identify oncogenes that encode tyrosine-protein kinase in human cancer cells (Kiyokawa *et al*, 1994; Peng and Cartwright, 1995; Goodman *et al*, 2001) and certain evidence suggests that oncogenes of the tyrosine kinase-type are involved in the tumorigenesis (Scrimgeour *et al*, 1997; Hung and Elliott, 2001) and immortalisation (McCormack *et al*, 1997; Vogt *et al*, 1989) of various types of human cancers. Aberrant expression of epidermal growth factor (EGF) receptors and HER2/*neu* proto-oncogene have been reported in primary lung cancer (Dazzi *et al*, 1989; Schneider *et al*, 1989; Kern *et al*, 1990). In general, it was proved that aberrant expression of oncogenes or EGF-receptors was associated with Tyr-phosphorylation (Reynolds *et al*, 1989; Takeshima *et al*, 1991) and that pTyr-proteins are deeply related to cell cycle regulation (Yaacov *et al*, 1991). Furthermore, increase of pTyr-proteins might correlate with the morphological transformation of tumour cells (Hamaguchi *et al*, 1988). Clinically, it was reported that overexpression of pTyr-proteins 100–130 kDa in molecular weight might predict shortened survival of lung cancer patients (Nishimura *et al*, 1996). Thus, a close relationship between tyrosine phosphorylation and biological characteristics of the malignant tumours has been speculated upon. However, there are few reports using clinical materials concerning pTyr-proteins in cancer cells.

In this context, we attempted to clinicopathologically evaluate the relationship between tyrosine phosphorylation and tumour characteristics using surgically resected materials of non-small cell lung cancer (NSCLC), and to discuss the clinical significance of pTyr-proteins in NSCLC.

## MATERIALS AND METHODS

### Preparation of surgically-resected tumours and normal peripheral lung tissues

Clinical materials were obtained from 96 patients with NSCLC, resected at the Department of Surgery, Tokyo Medical University from October 1997 to May 1999. All specimens were collected from cancerous tissues and normal peripheral lung tissues located far from the cancerous lesion in the same lobe. The cancerous tissue materials (10×10×3 mm in size) were collected from relatively near the margin of the primary lesions to avoid both necrosis and scar tissue. After tissue collection, inhibition of disphosphorylated kinases is immediately necessary. Therefore, tissue-materials were incubated in 1 mM orthovanadate solution at 200 mmHg for 5 min (Akimoto *et al*, 1998). After inhibition of disphosphorylated kinases, materials were acetone-fixed and paraffin-embedded (Sato *et al*, 1986). Sample preparation (the inhibition of disphosphorylated kinases) was started within 30 min after the surgical resection.

The 96 cases were diagnosed pathologically as adenocarcinoma (62 cases), squamous cell carcinoma (31 cases), and large cell carcinoma (three cases). Furthermore, adenocarcinoma and squamous cell carcinoma was histopathologically classified as well, moderately and poorly differentiated. Tumours were classified according to the histological subgroups recommended by the World Health Organization (Travis *et al*, 1999) and staged by the tumour–nodal involvement–metastasis (TNM) system according to the UICC TNM classification, fifth edition (Sobin and Wittekind, 1997).

### Immunohistochemical staining

The surgically resected specimens were stained immunohistochemically using an avidin-biotin peroxidase complex (ABC) method (Akimoto *et al*, 1998). We used a mouse monoclonal antibody clone 4G10 (VEC, NY, USA) (Druker *et al*, 1989; Cohen *et al*, 1990; Kanakura *et al*, 1991) for the detection of pTyr-proteins, and anti-proliferating nuclear antigen (PCNA) mouse monoclonal antibody PC10 (DAKO, Copenhagen, Denmark) for the detection of proliferating activity of tumour cells. We evaluated pTyr-proteins and PCNA using consecutive slices of tissues.

### Expression levels of the tyrosine phosphorylation and cell proliferation

Epithelial cells with cytoplasmic staining were judged as the pTyr-proteins-rich cells when pTyr-proteins were evaluated. Cases were classified into three groups according to the frequency of positive cells: high expression (*High*), more than 68% (mean±standard deviation (s.d)) positive cells; intermediate expression (*Int*), 9–68% (mean±s.d.) positive cells; and low expression (*Low*), less than 8% (mean±s.d.) positive cells.

Cell proliferating ability was evaluated using the PCNA labelling index of positive nuclear staining cells.

### Western blot and immunodetection of pTyr-proteins

Disphosphorylated kinases-inhibited materials of human lung cancer and normal peripheral lung were subjected to 7.5% SDS-polyacrylamide gel electrophoresis as described previously (Laemmli, 1970; Weiner *et al*, 1990; Kanakura *et al*, 1991), and proteins were transferred electrophoretically to Immobilon PVDF membrane (Millipore). PTyr-proteins on the membrane were immunodetected with monoclonal antibody (clone 4G10) and an enhanced chemiluminescence (ECL) western detection reagents kit (Amersham, Little Chalfont, Buckinghamshire, UK) (Kaufmann *et al*, 1987; Hoffman and Jump, 1989).

### Statistical analysis

Statistical analysis of the data was performed by Student's *t*-test. The Kaplan–Meier method was used to estimate the disease-free survival probability for each group. The log rank test was used to compare survival curves. Differences were considered statistically significant when the *P*-value was less than 0.05. Also, the influence of variables on postoperative disease-free survival was assessed using Cox's proportional hazard model. The following variables were used in the Cox regression: t-factor, n-factor, lymphatic invasion, vascular invasion and pTyr-protein expression levels. A value of less than 0.05 was considered to indicate a statistically significant difference.

## RESULTS

### pTyr-proteins expression

**Figure 1:**
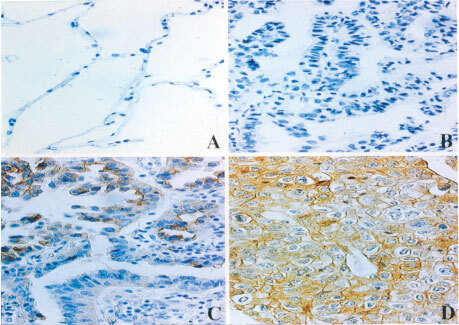
Immunohistochemical staining of phosphorylated tyrosine-containing proteins (pTyr-proteins) (**A**) Normal peripheral lung tissue; **B–D**: non-small cell lung cancer. (**B**) Low expression, (**C**) intermediate expression, (**D**) high expression.

The features of immunohistochemical staining are shown in Figure 1

. Cells with pTyr-proteins were detected in 71 cases (74.0%) out of the 96 NSCLC cases, but with various expression levels. On the other hand, only 13 (13.6%) normal peripheral lung tissues showed positive cells. Furthermore, 10 of these 13 cases possessed less than 20% positive cells.

**Figure 2:**
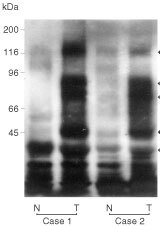
Detection of pTyr-proteins in human tissues of peripheral lung and lung cancer using Western blot analysis. We detected pTyr-proteins with various molecular weights. Arrowheads showed pTyr-proteins with approximately 120, 85, 75, 45 and 30 kDa in molecular weight. N: normal peripheral tissue; T: cancerous tissue. Case 1: Adenocarcinoma; Case 2: Squamous cell carcinoma.

Also, we detected pTyr-proteins in Western-blot analysis (Figure 2

). Cancerous tissues expressed pTyr-proteins showing various molecular weight (120, 85, 75, 45, and 30 kDa) in the cancerous tissues. On the contrary, a few pTyr-proteins were expressed in the normal peripheral lung.

Figure 3

**Figure 3:**
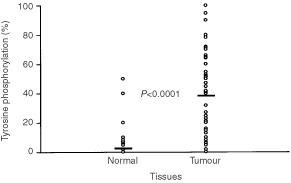
The relationship of pTyr between normal (mean±s.d.: 2.26±8.03) and tumour (mean±s.d.: 38.13±30.15) tissues was showed. The Student's *t*-test was used to estimate the tyrosine phosphorylation levels.

shows the expression levels of pTyr-proteins in the normal and malignant tissues. There was a statistically significant difference in pTyr-protein expression between normal and cancerous tissues (*P*<0.0001).

### Tyrosine phosphorylation and clinicopathological features

Table 1

**Table 1:**
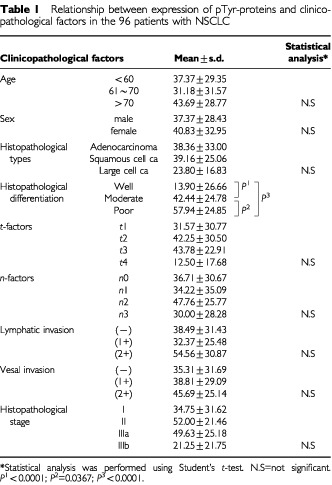
Relationship between expression of pTyr-proteins and clinicopathological factors in the 96 patients with NSCLC

shows the relationship between the expression levels of pTyr-proteins and clinicopathological factors. No statistically significant differences were found in any clinicopathological factors. However, there was a statistically significant difference among different degrees of histopathological differentiation of NSCLC (*P*<0.0001 between well and moderate differentiation, *P*<0.0001 between well and poor differentiation and *P*=0.0367 between moderate and poor differentiation).

### The relationship between expression levels of pTyr-proteins and cell proliferation

Figure 4

**Figure 4:**
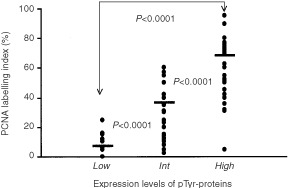
The relationship between pTyr expression levels and PCNA (mean±s.d.: p-Tyr(*Low*), 7.08±8.79; p-Tyr(*Int*), 36.74±19.31; p-Tyr(*High*), 68.57±11.20) was shown. The statistical analysis was performed using Student's *t*-test.

shows the relationship between the expression levels of pTyr-proteins and cell proliferating ability using the PCNA labelling index in NSCLC tissues. There was a statistically significant difference between each group (*P_(Low, High)_*<0.0001; *P_(Low, Int)_* <0.0001; *P*_(*Int, High*)_<0.0001).

### The expression levels of pTyr-proteins and the disease-free survival

**Figure 5:**
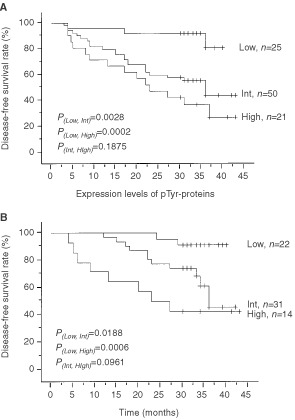
Actuarial curves for disease-free survival of patients with NSCLC according to the levels of pTyr-proteins. (**A**) All patients with NSCL; (**B**) Stage I-patients with NSCLC. The Kaplan–Meier method was used to estimate the survival distribution for each group. The log rank test was used to evaluate the equality of the survival curves.

Postoperative follow-up-periods in all cases were more than 25 months in this study. Figure 5

shows the disease-free survival curves for each expression levels of pTyr-proteins. There was a statistically significant difference between cases with low expression and intermediate expression (*P*=0.0028), and cases with low expression and high expression (*P*=0.0002) (Figure 5a). Only in stage I cases was there a statistically significant difference between cases with low expression and intermediate expression (*P*=0.0188), and cases with low expression and high expression (*P*=0.0006) (Figure 5b).

### The contribution to postoperative disease-free survivals

All variables (t-factor, n-factor, lymphatic invasion, vascular invasion and pTyr-protein expression levels) were included in a multivariate analysis by Cox proportional hazards model. In the stepwise analysis, n-factor, vascular invasion and pTyr-proteins expression level revealed statistical significance. PTyr-proteins expression levels were found to be an independent prognostic indicator as well as n-factor and vascular invasion (Table 2A

**Table 2:**
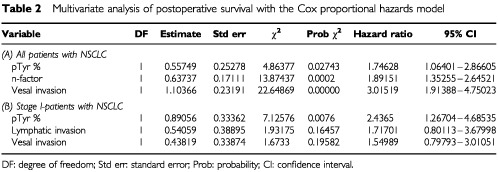
Multivariate analysis of postoperative survival with the Cox proportional hazards model

). In the investigation of only stage I-cases pTyr-protein expression levels were significantly related to disease-free survival. In the stepwise analysis no other variable revealed statistical significance (Table 2B).

## DISCUSSION

Primary lung cancer is an aggressive and highly malignant tumour frequently having an extremely miserable outcome. Even in lung cancers diagnosed as pathological stage I, approximately 30% relapse mainly due to distant metastasis. At present pathological staging based on TNM classification is the most reliable method to estimate patient prognosis, but the results are not satisfactory. Biological markers reflecting tumour characteristics might enhance evaluation of patient prognosis.

It is now known that carcinogenesis and tumour progression are related to the accumulation of mutations of oncogenes and tumour supressor genes (Fearon and Vogelstein, 1990; Sozzi *et al*, 1992; Sundaresan *et al*, 1992; Hirano *et al*, 1994). Nearly half of known oncogenes encode tyrosine kinases and are distributed throughout whole human cells. Yet, there are a few reports concerning the relationship between the expression of pTyr-proteins and human cancer (Hunter and Sefton, 1980; Shibamoto *et al*, 1994). According to a previous investigation using immunoprecipitation, the overexpression of tyrosine-phosphorylated 100–130 kDa proteins correlated with poor prognosis in lung cancer cases (Nishimura *et al*, 1996; Imaizumi *et al*, 1997). We also detected these 100–130 kDa pTyr-proteins by Western blot analysis of surgically resected materials of both normal and cancerous tissues of the lung (Figure 2). However, the high expression of these proteins is not always associated with high malignancy of lung cancer. Also, pTyr-proteins with more varied molecular weights, including lower molecular weights, were detected in malignant tissues. There is a possibility that the pTyr-proteins with relatively lower molecular weight may belong to the other kinds of tyrosine kinase including *src* family and its related proteins. Therefore, we attempted to immunohistohemically evaluate all pTyr-proteins in surgically resected NSCLC tissues as well as in normal peripheral lung tissues from the same lobe, but distant from the primary lesion.

In only 13 out of 96 (13.6%) normal peripheral lung tissues were pTyr-proteins detected, even at a low expression level. We have to emphasise that protein phosphorylation is essential for maintaining normal functions in normal cells, even if generally the expression levels are extremely low compared with the expression level in malignant neoplastic tissues. Indeed, we also detected very low levels of pTyr-proteins even in ciliated bronchial epithelium as well as alveolar cells (data not shown).

In lung-cancerous tissues various expression levels of pTyr-proteins were detected in 71 out of 96 (74.0%) lung cancer materials. Furthermore, as the degree of histopathological differentiation of cancerous tissues became poorer, the expression levels of pTyr-proteins increased. These results support the concept that overexpression of pTyr-proteins may be associated with malignant transformation and progression of lung cancer. We also obtained data indicating a positive relationship between elevations of pTyr-proteins and increased cell proliferation ability. Most growth factor receptors, including epidermal growth factor (EGF) receptor, platelet derived growth factor (PDGF) receptor and HER2/*neu* possesses tyrosine-kinase activity and have been reported to be closely associated with cell proliferation (Kokai *et al*, 1988; Rahimi *et al*, 1998; Tanaka *et al*, 1998; Liu *et al*, 2000). In this study, we evaluated cell proliferation immunohistochemically in terms of the labelling index of PCNA using the consecutive slice of the same materials used for evaluation of pTyr-proteins. A closely significant relationship between pTyr-proteins and PCNA was recognised (Figure 4). Therefore, our data supported that the mechanisms of the elevation of pTyr-proteins are probably related to cell growth though several pathways.

Also, a statistically significant difference in the expression levels of pTyr-proteins according to the level of histopathological differentiation was recognised. At present, histopathological differentiation is diagnosed by experienced pathologists mainly based on histopathological and cellular structures. In general, poorly differentiated tumours show large differences in cellular shapes and sizes and loss of histological structures. Even though histopathological differentiation is one of the most important factors reflecting tumor malignancy, the criteria of histopathological differentiation are largely subjective. α- and β-catenins are essential for tight cell-cell adhesion through e-cadherin, because the cadherin-catenine complex combines with actin filaments. The deletion of these molecules causes tumour cells not only to lose the function of cell-cell adhesion but to change cellular formation (Shimoyama *et al*, 1992). In addition, it is already known that the phosphorylation of β-catenins induce dysfunction of e-cadherin (Matsuyoshi *et al*, 1992; Hamaguchi *et al*, 1993). Therefore, there is a possibility that phosphorylation may be related to morphological alterations.

The clinical impact of this study may be to clarify the significant relationship between the expression of pTyr-proteins and survival probability after surgery. Our findings indicate that overexpression of pTyr-proteins is associated with poor disease-free survival in spite of the absence of correlation with clinicopathological factors. At the same time, Cox regression multivariate analysis indicated that vascular invasion, n-factor and pTyr-proteins were unfavourable factors. Furthermore, this analysis showed pTyr-proteins overexpression to be only one significant prognostic-factor among stage I NCSLC cases. In this context, there is a high possibility that pTyr-protein overexpression must be deeply associated with malignant biological characteristics, and that several mechanisms of phosphorylation in cancerous cells are responsible for poor prognosis in the patients with NSCLC. As new therapeutic strategies, adjuvant therapy seems to be needed for stage I-cases with pTyr-proteins overexpression. In addition, world-wide clinical trials using a tyrosine kinase inhibitor seems to be obtaining favourable results. When this kind of medicine will be used clinically in the near future, the evaluation of expression levels of pTyr-proteins might be valuable for the prediction of effectiveness of tyrosine kinase inhibitors.

This study suggests that tyrosine-phosphorylation is related to histopathological differentiation, cell proliferating ability and poor prognosis. According to previous reports, a few independent survival markers (Bcl-2 for longer survival, and mutant-p53 for shorter survival) were shown (Ohsaki *et al*, 1996; Moldvay *et al*, 2000). Our study did not focus on the evaluation of a specific molecule. The evaluation of pTyr-proteins reflected the expression levels and activities of various kinds of tyrosine-kinase, cell proliferation ability and cell to cell adhesion indirectly and comprehensively. In addition, pTyr-proteins expression levels were independent of pathological TNM-staging. Therefore, we conclude that evaluation of pTyr-proteins is valuable to more fully understand the biological characteristics of tumour cells, and we believe that it may be valuable for constructing the most appropriate therapeutic strategy.
